# Electrochemical Genosensing of Circulating Biomarkers

**DOI:** 10.3390/s17040866

**Published:** 2017-04-14

**Authors:** Susana Campuzano, Paloma Yáñez-Sedeño, José Manuel Pingarrón

**Affiliations:** Departamento de Química Analítica, Facultad de CC. Químicas, Universidad Complutense de Madrid, E-28040 Madrid, Spain; yseo@quim.ucm.es

**Keywords:** circulating biomarkers, electrochemical genosensors, cancer, bacterial and viral infections, neurodegenerative diseases

## Abstract

Management and prognosis of diseases requires the measurement in non- or minimally invasively collected samples of specific circulating biomarkers, consisting of any measurable or observable factors in patients that indicate normal or disease-related biological processes or responses to therapy. Therefore, on-site, fast and accurate determination of these low abundance circulating biomarkers in scarcely treated body fluids is of great interest for health monitoring and biological applications. In this field, electrochemical DNA sensors (or genosensors) have demonstrated to be interesting alternatives to more complex conventional strategies. Currently, electrochemical genosensors are considered very promising analytical tools for this purpose due to their fast response, low cost, high sensitivity, compatibility with microfabrication technology and simple operation mode which makes them compatible with point-of-care (POC) testing. In this review, the relevance and current challenges of the determination of circulating biomarkers related to relevant diseases (cancer, bacterial and viral infections and neurodegenerative diseases) are briefly discussed. An overview of the electrochemical nucleic acid–based strategies developed in the last five years for this purpose is given to show to both familiar and non-expert readers the great potential of these methodologies for circulating biomarker determination. After highlighting the main features of the reported electrochemical genosensing strategies through the critical discussion of selected examples, a conclusions section points out the still existing challenges and future directions in this field.

## 1. Introduction

Early diagnosis and treatment of any disease reduces its severity and possible complications, playing an important role in successful treatment. The presence of various disease-signaling biomarkers in non- or minimally invasively collected biofluids accurately reflect normal and disease states in humans. Therefore, there is a great demand for developing new strategies for the reliable detection of circulating biomarkers due to the attractiveness of these approaches for non-invasive clinical diagnosis and prognosis and the compatibility with point-of-care (POC) applications. These strategies should improve clinical diagnosis, management, and treatment and accelerate decision-making in emergency situations (through rapid determination of parameters critical in time such as cardiac markers and blood metabolites). This demand of sensors especially tailored for the particular requirements of real-time analysis in physiological samples has driven the development of POC tests (POCT) able to provide non-trained personnel with clinical results within a few minutes in settings different from those of normal healthcare services [1]. In this context, electrochemical genosensing can be considered particularly well suited for this purpose as a consequence of its inherent high sensitivity and selectivity, simplicity, availability, affordable instrumentation, feasibility for miniaturization and suitability for decentralized applications. 

**Figure 1:**
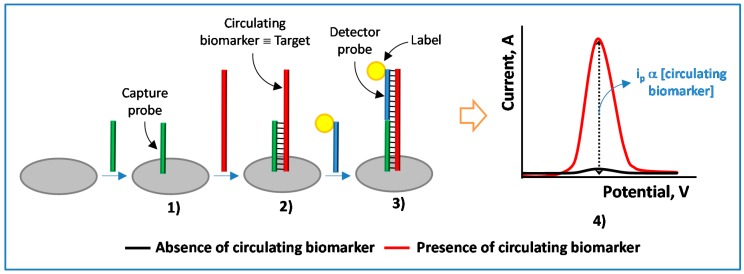
Schematic display of a general nucleic acid-based sensor for the determination of a particular target involving immobilization of a specific capture probe on the electrode surface (**1**); specific hybridization of the immobilized capture probe with the target sequence (**2**) and of the captured target with the label-conjugated detector probe (**3**) and electrochemical detection of the hybridization reactions by differential pulse voltammetry of the label attached to the detector probe (**4**).

Electrochemical genosensing seeks to exploit the great potentiality of coupling specific and efficient affinity reactions involving nucleic acids with electroanalytical techniques. This combination typically allows the development of inexpensive and rapid methods to determine analytes in a highly sensitive and selective manner, in a broad context facing a wide variety of analytical problems in biomedical research, drug discovery, environment, food, process industries, security and defense and particularly relevant nowadays in clinical diagnosis. Although electrochemical nucleic acid-based sensors exploit a range of distinct chemistries, they all take advantage of the nanoscale interactions (mainly hybridizations, ligations or conformational changes) between the target present in solution, the recognition layer, and the electrode surface, allowing their electrochemical detection using inexpensive analyzers [2]. As a general example, Figure 1 shows a schematic display of the main steps involved in an electrochemical DNA sensor for the detection of a particular target using a sandwich hybridization format and electrochemical detection (by differential pulse voltammetry) of the redox label with which the detector probe has been conjugated. Moreover, the growing research in advanced nanomaterials has impressively impacted also this field, by providing a large variety of novel, versatile and rationally designed nanostructured supports for transduction, signal generation and amplification purposes [3]. Indeed, the specific and efficient interactions involved in electrochemical genosensing can be detected by using various electrochemical strategies that exploit the redox reactions of reporters or enzymes, direct or catalytic oxidation of nucleic acid bases and electrochemistry of nanoparticles, conducting polymers (CPs) and quantum dots. In parallel with the major advances in nanotechnology, the wide variety of commercially available nucleic acids conjugated on demand with a variety of tags depending on the required needs, the versatility of design and modification offered by electrochemical substrates and the excellent performance and variability of powerful electrochemical techniques have also played major roles in the outstanding capabilities demonstrated by electrochemical biosensors. The aim of this review article is to shed useful insights into the latest advances, current trends and challenges and future prospects in the exciting field of electrochemical genosensing focused on the determination of circulating biomarkers.

## 2. Circulating Biomarkers

Circulating biomarkers are biomolecules that can be objectively measured in a wide variety of body fluids (blood, urine, saliva, amniotic fluid, breast milk, bronchoalveolar lavage, cerebro-spinal fluid, semen, pleural effusion, ascites, etc.) and evaluated as indicators of normal or abnormal processes or conditions or diseases and pharmacologic responses to therapeutics [4]. Concentration levels of these molecules are representative of changes occurring in the basic cell regulatory functions and cellular physiology, such as cell division or contact inhibition, and may vary from their normal levels in case of diseases and disorders [5]. Circulating biomarkers can derive from various molecular origins, including small molecules such as glucose or cholesterol, DNAs (i.e., specific mutation, translocation, amplification in characteristic genes such as *BCRA1*, *KRAS*, cell-free DNAs, RNAs (mRNAs and miRNAs), proteins (i.e., hormones, antibodies, oncogenes, or tumor suppressors), exosomes and tumor cells. These circulating biomarkers in general can be categorized into different groups according to their potential usage [4,6]:

- Risk screening biomarkers, used to determine disease susceptibility of an individual,
- Prognostic biomarkers which provides information on the probability of the disease in an untreated individual,
- Predictive biomarkers, particular proteins or genes indicating sensitivity or resistance to a specific therapy, and
- Diagnostic and disease monitoring biomarkers, used to aid the diagnosis and monitoring the progression of disease.

Since different diseases (cancer, bacterial and viral infections and neurodegenerative diseases) are known to produce or alter circulating biomarkers levels, they have the potential to be used in understanding the pathogenesis of disease prognosis and for diagnostic purposes, as well as in the development of new therapeutic treatment regimens [7]. Therefore, sensitive and rapid analytical methods are very important for the accurate determination of circulating biomarkers in liquid biopsy samples. In order to avoid false positives and diagnostic ambiguity, which can arise from population variations in expression of a single biomarker in certain diseases such as cancer and/or from the lack of specificity to a particular disease of the target biomarker, simultaneous measurements of a panel of biomarkers are typically required [5,8]. These parallel measurements of groups of biomarkers hold enormous potential for early disease detection and personalized therapy [9].

Although genomic (e.g., DNA sequencing, PCR amplification detection, DNA microarray assay) and proteomic (conventional enzyme linked immunosorbent assays, ELISAs) methods are being extensively developed for circulating biomarkers determination, they currently involve labor-intensive, high-cost, and time-consuming processes which demanded skilled people, and have limited multiplexing capabilities, all in all significantly limiting their applications to diagnostics at POC [6]. Therefore, there is an urgent need to develop portable, simple, rapid, inexpensive tools for accurate determination of circulating biomarkers, which could be readily implemented in decentralized and resource-limited settings to overcome the shortcomings of conventional methodologies.

## 3. Electrochemical Genosensing of Circulating Biomarkers

Nowadays, electrochemical nucleic acid biosensing (genosensing), based on the use of sequence-specific probes as a biorecognition element and electrochemical transduction to convert nucleic acid hybridization into a detectable signal, has demonstrated great promise to address the deficiencies of conventional technologies for determination of circulating biomarkers associated with pathogenic, genetic, emerging and infectious diseases and inheritable and non-communicable diseases such as cancer and cardiovascular diseases [10]. Electrochemical genosensing combines the inherent selectivity provided by sequence-specific nucleic acid hybridization or conformational change with the unique advantages of electrochemical sensing technology, such as high sensitivity and selectivity, low cost, easy handling, simple instrumentation, rapid responses, miniaturization ability and easy integration into portable platforms and feasibility for multiplexing [3,6,10,11,12,13,14].

Electrochemical genosensors involve the immobilization of a specific nucleic acid recognition layer, able to recognize selectively a biomarker of genetic or protein nature, onto the electrode surface and the examination of electrochemical response before and after the occurrence of the bioaffinity reaction with the target analyte. Different strategies have been employed for electrochemical detection of DNA-target analyte interaction: direct and sandwich hybridization assays using enzyme or redox indicators as labels, assays based on DNA conformational changes and measurement of the electron transfer variation before and after the recognition [15].

The main bioreceptors used in electrochemical genosensing of circulating biomarkers include specific single-stranded (ss) DNA (both linear and hairpin probes) and RNA sequences, aptamers and peptides. Aptamers are in vitro selected ss-DNA or RNA molecules capable of binding a wide range of nucleic and non-nucleic acid molecules (proteins, viruses, bacteria or whole cells) with high affinity and specificity [16,17,18]. In solution aptamers are folded into a 3D structure containing binding site for the target. The affinity of aptamers to their targets is in the micromolar to the subnanomolar range and can therefore be comparable and in certain cases even better than those of antibodies for the same targets. Moreover, in comparison with antibodies, aptamers are more stable and flexible and since they are selected in vitro their sequence can be reproduced with high accuracy. In addition, aptamers can be chemically modified by various labels (redox, quantum dots and nanoparticles), allowing them to be immobilized at various surfaces and developing a wide variety of assay formats and amplification strategies [18].

Apart from these high affinity bioreceptors, the advances in the use of magnetic beads (MBs), screen-printed electrodes (SPEs) and nanomaterials have played major roles in improving the analytical performance of electrochemical genosensors, in their miniaturization and in their production costs reduction. 

The use of MBs substantially improved electrochemical genosensing in terms of enhanced sensitivity, reproducibility, and reduced assay turnaround [13]. MBs provide a large surface area for bioreceptor immobilization and bioreactions, serving as effective solid supports for the straightforward isolation, purification, and pre-concentration of target analytes using an external magnetic field. The MB-based genosensing approaches perform the hybridization and transduction steps on different surfaces (the MBs and unmodified electrodes, respectively), which eliminates the major problems associated with nonspecific adsorptions of non-target sample components, enhancing considerably the assay specificity and sensitivity.

Low cost and mass produced SPEs have been extensively employed for developing novel electrochemical genosensors providing advantages such as portability, miniaturization and low sample consumption, contributing to the great potential they have in POC tests. Other interesting characteristics of SPEs are their versatility of design which allows customizing their production and modification for special applications and multiplexed analysis. Moreover, the planar nature of the SPEs makes easy the modification of their surface, even in a mass-producible way using an automatic dispenser, and their coupling with MBs to develop MBs-based electrochemical genosensors [4].

Since the performance of electrochemical genosensors depends strongly on the amount and stability of the nucleic acid probes immobilized at the surface of the sensing device, the incorporation of a wide variety of unique nanomaterials such as graphene, graphene oxide, mesoporous nanomaterials, gold nanoparticles (AuNPs), gold nanorods (AuNRs) and multi-walled carbon nanotubes (MWCNTs), with large surface area, abundant binding points, favorable catalytic activity, conductivity and biocompatibility [19], into electrochemical genosensors has contributed to maximize their detection capabilities. The coupling of nanomaterials with electrochemical genosensors has demonstrated to improve the selectivity and the signal-to-noise ratios, thus increasing sensitivity and lowering the detection limits (LODs), minimize interference from biological matrices and prolong their stability meeting the demands for detection of extremely low concentrated circulating biomarkers [20].

Within this interesting context, this review discusses briefly the potential of electrochemical genosensors for the determination of circulating biomarkers and highlights some relevant approaches, classifying them regarding the type of biomarker determined. We will focus only in methods reported in the last five years which have been evaluated for circulating biomarkers determination in liquid biopsy samples, in connection to diseases of great current relevance such as cancer, neurodegenerative diseases and bacterial and viral infections. Main bottlenecks involved and possible research directions are also pointed out.

### 3.1. Electrochemical Genosensing of Cancer Biomarkers

Chen et al. [21] developed a very sensitive and selective electrochemical sensor based on nuclease-assisted target recycling and DNAzyme for determination of oral cancer overexpressed 1 (ORAOV1)-related DNA species. The authors used a thiolated signaling probe (Sp), self-assembled together with 6-mercaptohexanol (MCH) on a gold disk electrode, which comprised a target recognition sequence containing a nicking endonuclease recognition sequence and a G-rich sequence. The duplex formed in the presence of the perfectly matched target was cleaved by the specific endonuclease. The target DNA dissociated from the Sp was then able to hybridize with a new un-nicked Sp remaining only the G-rich sequence part on the electrode surface. After completion of the strand-scission cycle and upon addition of hemin, the G-rich sequence can form the G-quadruplex-hemin DNAzyme and catalyze the enzymatic reduction of H_2_O_2_ mediated by 3,3′,5,5′ tetramethylbenzidine (TMB). The as-developed amperometric genosensor showed a linear correlation with the target DNA concentration over the 0.08–8.0 fM range, a LOD of 0.02 fM, a good discrimination against single-base mismatches and demonstrated good results in the analysis of artificial saliva samples. A homogeneous immobilization-free electrochemical genosensing method was developed by Tan et al. to determine also ORAOV1-related DNA species [22]. The strategy involved the use of a methylene blue (MB) labeled substrate strand and signal amplification of nicking endonuclease assisted target recycling at negative charged indium-tin oxide (ITO) electrode. By measuring the reduction signal of MB using DPV, this method detected as low as 0.35 pM of the target DNA and was applied to the analysis of spiked saliva samples.

Wei’s group developed a multiplexed electrochemical sensor to detect mutations in the epidermal growth factor receptor (*EGFR*) gene directly in saliva samples [23]. The biosensing strategy involves the detection of the target biomarkers released from saliva by applying an electric field and using specific sandwich hybridization assays performed onto a conducting polymer-modified electrochemical array of 16 gold electrodes. The target DNAs were sandwiched between capture probes copolymerized with pyrrole (Py) onto the bare gold electrodes and detector probes labeled with fluorescein isothiocyanate (FITC). Upon incubation with an HRP-conjugated anti-FITC antibody, the hybridization efficiency was followed by measuring the cathodic current obtained by chronoamperometry at −0.2 V vs. Au electrodes in the presence of H_2_O_2_ and TMB. The usefulness of the multiplexed genosensing platform was demonstrated by detecting *EGFR* mutations in saliva samples of patients with non-small cell lung carcinoma (NSCLC).

A DNA sensor for *BRCA1*, a breast cancer-related gene detection was proposed by Rasheed et al. [12] using a graphene oxide-modified glassy carbon electrode (GO/GCE). The method implied a sandwich hybridization strategy using amino terminated capture and detector probes, covalently immobilized though 1-ethyl-3-(3-dimethylaminopropyl)carbodiimide/*N*-hydroxysuccinimide (EDC/ NHS) chemistry on GO/CGE and *O*-(3-carboxypropyl)-*O*′-[2-(3-mercaptopropionyl-amino)ethyl]-polyethylene glycol (CPEG)-modified AuNPs, respectively. By measuring chronoamperometrically at +1.1 V vs. SCE the anodic current of conjugated AuNPs, a linear response was found with the logarithmic target DNA concentration in the 1 fM to 1 nM range and a 1 fM LOD was reported.

Pingarrón’s group [24] developed an electrochemical DNA biosensor for detection of a target DNA sequence of the *p53* tumor suppressor gene utilizing SPCEs modified with a reduced graphene oxide-carboxymethylcellulose hybrid nanomaterial. The modified electrodes were used as covalent immobilization platforms for a selective 33 nts DNA hairpin-forming capture probe with biotin and amine modifications at the 5′ and 3′ ends, respectively. A streptavidin-peroxidase (Strep-HRP) conjugate was employed as electrochemical indicator. Hybridization was monitored by amperometry using TMB as redox mediator and H_2_O_2_ as enzyme substrate. These disposable biosensors provided a LOD of 2.9 nM for the synthetic target without any target or signal amplification process as well as complete discrimination towards a single base mismatch in untreated and undiluted spiked human serum and saliva samples. The same group developed a MB-based genosensing approach for the determination of mRNA associated to interleukin-8 (IL-8) mRNA [25]. The method was based on the hybridization of the target biotinylated oligonucleotide onto Strep-MBs modified with a specific biotinylated hairpin probe, labeling with a Strep-HRP polymer and amperometric detection at SPCEs using the H_2_O_2_/hydroquinone (HQ) system. This approach showed a LOD of 0.21 nM and successful recoveries in raw undiluted human saliva samples.

Huang et al. [26] developed an electrochemical DNA biosensor for very sensitive determination of human papillomavirus (HPV), associated with 93–100% of worldwide invasive carcinomas [26,27,28], by immobilizing a specific capture probe on a GCE modified with graphene/Au nanorods/polythionine. A sandwich hybridization format was used for the determination of the target DNA (a 34-base fragment specific from *HPV-16* gene) by means of two auxiliary probes (AP1 and AP2) for amplification purposes via a long-range self-assembled DNA nanostructure. The DPV signal of [Ru(phen)_3_]^2+^ intercalated in the long dsDNA structure was used to determine the target DNA concentration. This genosensor displayed excellent performance for HPV DNA determination with a linear range between 1.0 × 10^−13^ and 1.0 × 10^−10^ M and a LOD of 4.03 × 10^−14^ M and was successfully applied to the analysis of serum samples.

MicroRNAs (miRNAs) are nowadays considered reliable non-invasive molecular biomarkers linked to the development of human cancers, cardiovascular diseases, and viral infections. Currently, electrochemical nucleic acid-based biosensing strategies for miRNAs determination involving direct [29,30] or competitive [31,32] hybridization assays, the use of novel affinity biosensors based on the specific recognition of RNA-RNA [33,34,35,36] or DNA-DNA [37,38] duplexes, and different amplification strategies [39,40,41,42,43,44,45] provide attractive alternatives for their implementation in the clinical routine able to overcome the limitations of conventional quantification strategies. Table 1 summarizes the main characteristics of electrochemical genosensors developed and applied successfully for the determination of cancer-related circulating miRNAs in biofluids.

### 3.2. Electrochemical Genosensing of Neurodegenerative Diseases Biomarkers

Miodek et al. [46] developed an electrochemical aptasensor for determination of human cellular prions (PrPC) proteins, responsible for the transmissible spongiform encephalopathies (TSEs), a group of fatal neurodegenerative diseases including Creutzfeldt-Jakob disease in human and spongiform encephalopathy in animals [47]. The genosensing platform involved immobilization of a specific biotinylated aptamer through streptavidin binding to a biotinylated scaffold. The scaffold was prepared by covalent immobilization of polyamidoamine (PAMAM) G4 dendrimer to MWCNTs adsorbed on the surface of a gold electrode and subsequent covalent attachment of the redox marker ferrocene (Fc) modified with two phtalamido groups (Fc(NHP)_2_) to the PAMAM G4 and of biotin hydrazide on the remaining phtalymidyl esters of the Fc moieties. The peak current values measured by cyclic voltammetry of Fc incorporated between the dendrimer and aptamer interlayer decreased with the PrPc concentration due to a slower electron transfer resulting from the permeability decrease of the sensing layer after attachment of the prion proteins to the surface. The feasibility of this approach, with a wide linear detection linear range (1 pM–10 μM) and a LOD of 0.5 pM, was demonstrated for detection of PrPC in spiked blood plasma samples.

**Figure 2:**
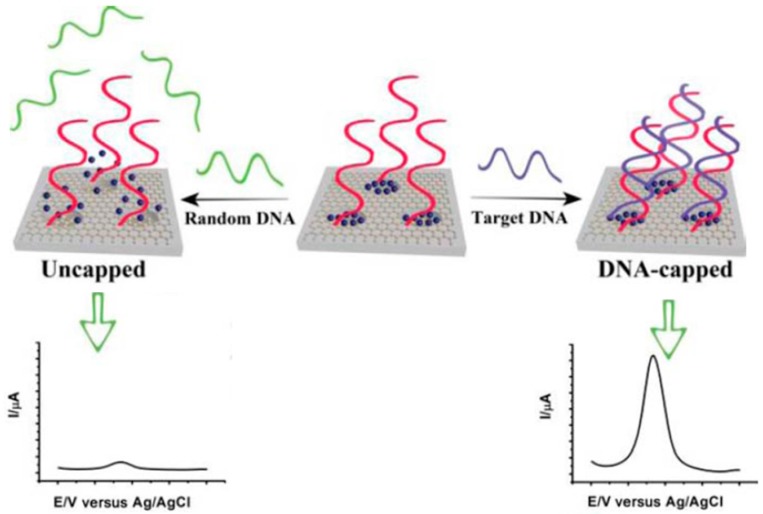
Schematic display of the target-responsive encapsulation nucleic acid-based electrochemical biosensor functioning and the DPV signals obtained in the presence and in the absence of the target DNA. Reprinted and adapted from [48] with permission.

An ultrasensitive electrochemical detection system using a target-responsive encapsulation assay, graphene and mesoporous materials (MSGNs) multifunctional hybrids and ss-DNA probes as the pore-caps was developed by Wu et al. [48]. The approach is based on the loading of electroactive molecules (MB and Fc) on MSGNs and the locking of the pores by target-induced conformational change of the tailored DNA caps. The specific DNA probes change their conformation closing pores only in the presence of the target analytes and therefore provoked an increased electroanalytical signal of the loaded electroactive molecules measured by DPV (see Figure 2). This scaffold allows the incorporation of various redox-active molecules into the mesopores and to be modified by different nucleic acid bioreceptors, which provides a high versatility and easy multiplexing ability to the approach. The usefulness of the biosensor was assessed by determining DNA sequences correlated to Alzheimer, thrombin and ATP in serum samples.

Same group developed a similar approach but using a novel ratiometric and on-off signaling mode for detection of the mutated *apolipoprotein E* gene associated with Alzheimer’s disease using graphene and mesoporous hybrid nanomaterials (GSHs)-modified GCE [49]. The GSHs, which served as nanoreservoirs for the electro-active molecules (such as MB), were covalently conjugated with ferrocene carboxylic acid, used as inner reference molecule for indicating the amount of GSHs used. While in the absence of target DNA a duplex DNA probe connected to the surface of GSHs prevented the leakage of loaded caged electrochemical reporter (MB), the presence of the target DNA induced the nanopores opening which led to the release of MB molecules and hence to a smaller signal. The ratiometric output signal was obtained by calculating the ratio of peak current measured by DPV of the signal-indicator (MB) and built-in control (Fc). 

### 3.3. Electrochemical Genosensing of Viral Infections Biomarkers

**Figure 3:**
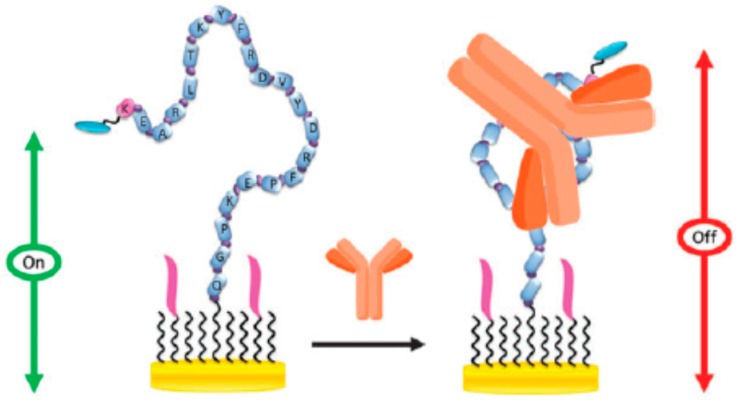
Electrochemical biosensor for HIV antibodies using a specific peptide and short DNAs as diluents. In the presence pf HIV antibodies the surface mobility of the immobilized peptide probe is limited and the MB current measured by ACV decreased. Reprinted from [50] with permission.

Interesting methods involving the use of specific DNA and peptide sequences have been reported recently for electrochemical genosensing of viruses and autoantibodies specific to them. An electrochemical peptide-based sensor for HIV antibodies detection was developed by McQuistan et al. [50] by self-assembling a specific thiolated peptide labeled with MB as electrochemical indicator, and short thiolated DNAs as anti-fouling diluents on gold electrodes (Figure 3). The recognition between the peptide probe and the antibody damped the probe flexibility and this limited surface mobility was followed by the decrease in the MB reduction peak obtained by alternating current voltammetry (ACV). Results reported by the authors demonstrated the major role of the short DNA diluents used to prevent surface fouling, a LOD of 1 nM and promising applicability to perform the determination of HIV antibodies in spiked 10% saliva samples. 

Oliveira et al. [51] proposed a method for the detection of DNA sequences specific of dengue virus serotype 3 (DENV-3). In this genosensing approach, a specific 22-mer DNA probe was adsorbed onto a pencil graphite electrode and DPV of the guanine oxidation signal was used for electrochemical analysis. This sensor allowed achieving a linear range from 10 to 100 nM, a LOD of 3.09 nM and selective detection of target sequences both in buffer and spiked human serum samples.

A highly sensitive impedimetric biosensor for detecting human norovirus by assembling a thiolated affinity peptide on a gold electrode was also developed [52]. This impedimetric genosensing approach showed a linear quantitative response to a wide range of norovirus concentrations (10–10^7^ copies mL^−1^) and LODs of 99.8 nM for recombinant noroviral capsid proteins (rP2) and 7.8 copies mL^−1^ for real norovirus from diarrheal patients. Results presented demonstrated also the feasibility of this method to perform the determination in fetal bovine serum.

### 3.4. Electrochemical Genosensing of Bacterial Infections Biomarkers

Wu et al. [53] achieved very sensitive chronoamperometric determination of *Escherichia coli* (*E. coli*) by preparing a ternary surface monolayer composed of a thiolated capture probe (specific for the *E. coli* 16S rRNA), MCH and dithiothreitol (DTT) onto a photolithography prepared 16-sensor Au electrode array. Using a sandwich hybridization assay involving fluorescein (FITC)-labeled detector probes, recognition by an HRP conjugated–anti-FITC antibody and chronoamperometric detection with the TMB/H_2_O_2_ system, the electrochemical genosensor provided absolute LODs of 40 zmol for the synthetic target DNA and only 1 colony forming unit (CFU) *E. coli* per sensor. Moreover, the applicability and specificity of the biosensor were evaluated in the analysis of real uropathogenic clinical isolates.

**Figure 4:**
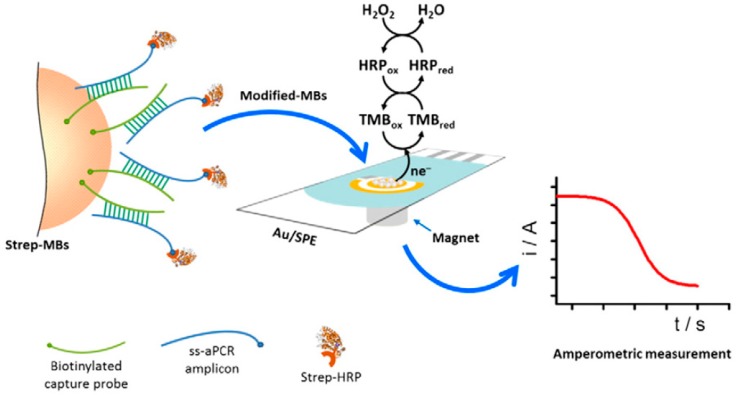
Schematic display of the MBs-based amperometric DNA sensor developed for *S. pneumoniae* determination through the detection of the ss-aPCR amplicon generated from a specific fragment of *lytA* gene coding sequence by performing daPCR directly in bacterial cultures. Reprinted and adapted from [55] with permission.

A disposable amperometric MBs-based DNA sensor coupled to asymmetric PCR (aPCR) was developed for the detection of *Streptococcus pneumoniae* (*S. pneumoniae*) [54]. This method relied on the selective hybridization of specific biotinylated capture DNA probe, complementary to a specific region of the pneumococcal *lytA* gene. The biotinylated synthetic target or the predominantly 235-base single-stranded (ss) amplicon generated by direct asymmetric PCR (daPCR) from bacterial cultures were hybridized onto Strep-MBs modified with a specific biotinylated DNA probe. After labeling of the biotinylated duplex formed at the Strep-MBs with Strep-HRP, amperometric detection was performed upon addition of H_2_O_2_ at tetrathiafulvalene (TTF)-Au-SPEs (Figure 4). LOD values of 5.1 and 1.1 nM for the 20-mer synthetic target DNA and the ss-aPCR amplicon were achieved, respectively. Moreover, daPCR amplicons could be obtained with as few as 2 CFUs of *S. pneumoniae*. Furthermore, this methodology demonstrated a clear discrimination against *Streptococcus mitis* (a closely related streptococcus) and between blood and urine samples non-inoculated and inoculated with the target bacteria at a very low concentration (10^3^ CFUs mL^−1^). The DNA sensor was successfully validated with 109 clinical samples of diverse origins providing both sensitivity and specificity of around 90% [55].

Yamanaka et al. [56] designed an electrochemical strategy to quantify *Porphyromonas gingivalis* (*Pg*), a bacterium causing periodontal disease, in saliva. The strategy was based on mixing bisbenzimidazole trihydrochloride as electrochemical intercalator with the amplicons obtained by direct PCR (dPCR). The indicator peak current, measured by linear sweep voltammetry (LSW), decreased in the presence of amplicons over the 1–10^4^ cells range due to the indicator slower diffusion after intercalation into the amplified DNA. The results achieved in the analysis of saliva samples confirmed that the quantitative detection of direct PCR-amplified products from *Pg* clearly reflect the periodontal disease degree of and the age-dependence.

A rapid urine test for detection of urogenital *Schistosomiasis* was developed by Mach et al. [57]. The biosensor relied on sandwich hybridization of the *Schistosoma haematobium* (*S. haematobium*) 16S rRNA using a specific thiolated DNA capture probe self-assembled onto a gold electrode and a detector probe labeled with fluorescein (FITC). Upon incubation with a specific HRP conjugated–anti-FITC antibody, the hybridization reaction was monitored by chronoamperometry in the presence of TMB/H_2_O_2_. The DNA sensor allowed detecting 0.53 ng mL^−1^ total RNA isolated from *S. haematobium* egg and approximately 30 *S. haematobium* eggs per mL of human urine.

**Figure 5:**
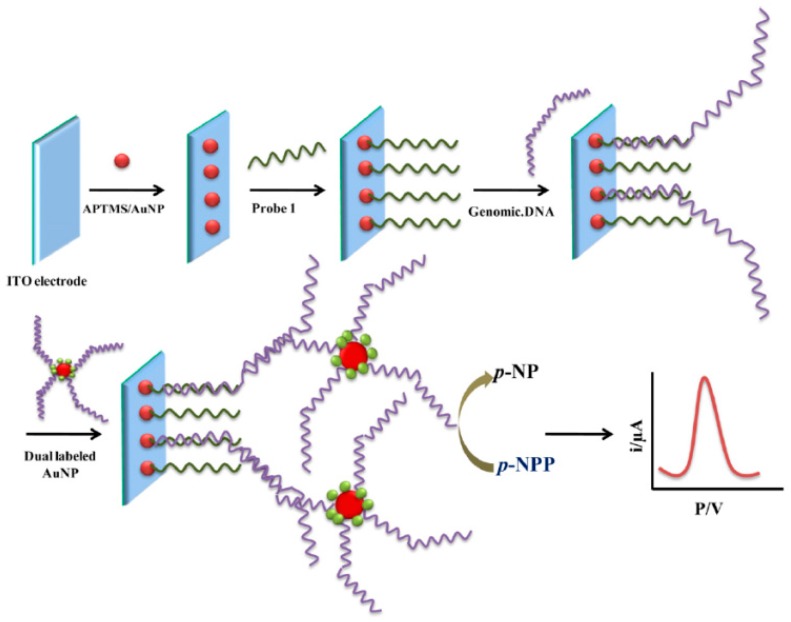
Schematic display of the electrochemical DNA biosensor developed for *Mycobacterium* sp. genomic DNA detection using a specific probe immobilized on an ITO electrode and AuNPs modified both with detector probes and the enzyme alkaline phosphatase (AP). Reprinted from [58] with permission.

Thiruppathiraja et al. [58] developed a sandwich-based DNA biosensor for *Mycobacterium* sp. genomic DNA detection using dual labeled-AuNPs for amplification purposes. The target DNA is sandwiched between a specific probe immobilized on a ITO electrode modified with a (3-aminopropyl)trimethoxysilane (APTMS) SAM and the detector probes immobilized together with the enzyme alkaline phosphatase (AP) on AuNPs (Figure 5). The electrochemical detection was performed by DPV using *p*-nitrophenol phosphate as enzyme substrate. The method allowed a LOD of 1.25 ng mL^−1^ genomic DNA and was successfully evaluated in clinical sputum samples.

Other electrochemical biosensor for *M. tuberculosis* [59] was prepared by covalent immobilization of an amino-terminated DNA probe (specific to the *rpoB* gene, the gene most commonly mutated in rifampicin resistant bacteria [60,61]) and Fc(NHP)_2_ onto a new composite material prepared by electrochemical assembly of PAMAM, polypyrrole (pPy) and MWCNTs onto a gold electrode. In this design, MWCNTs coated with pPy were prepared by wrapping the polymer film on MWCNTs during electrochemical polymerization of Py on the gold electrode. Subsequently, the MWCNTs-pPy layer was modified with PAMAM dendrimers of fourth generation (PAMAM G4) with covalent bonding by electro-oxidation. The hybridization was followed by measuring SW voltammetric signals of Fc. This biosensing strategy was shown to be suitable for the determination of amplicons obtained from clinical samples and for discriminating single nucleotide polymorphism sequences, which are responsible for resistance to rifampicin.

**Figure 6:**
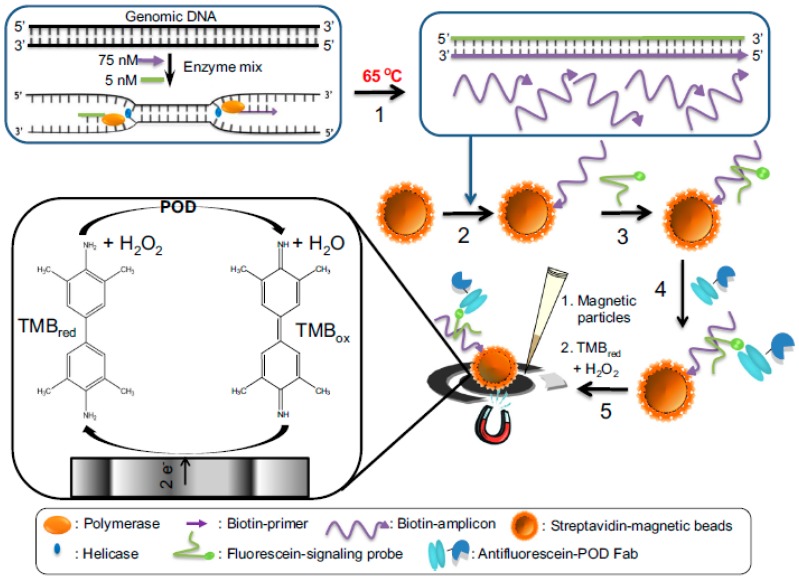
Schematic representation of the steps involved in the *M. tuberculosis* determination using an electrochemical genomagnetic assay coupled to HDA. The biotinylated ss-amplicon resulting from the HDA was immobilized onto the surface of Strep-MBs and further hybridized with an FITC-detector probe and labeled with an HRP-antiFITC antibody. Reprinted from [62] with permission.

Lobo-Castañón´s group developed also an attractive electrochemical genomagnetic approach by combining the use of MBs and chronoamperometric detection at SPCEs with asymmetric helicase-dependent DNA amplification (HDA) to amplify an 84-base-long ss-DNA fragment from the insertion sequence IS6110 characteristics of *M. tuberculosis* [62]. The method involved the immobilization of the biotinylated ss-amplicon onto the surface of Strep-MBs, the hybridization of the captured amplicon with an FITC-detector probe and subsequent labeling with an HRP-conjugated antiFITC antibody (Figure 6). The electrochemical transduction was performed upon capturing magnetically the modified MBs onto the SPCE surface by chronoamperometry and the H_2_O_2_/TMB system. The method allowed obtaining a linear range from 1 aM to 1 fM and LOD of 0.5 aM for the synthetic target DNA determination in less than 4 h. A year later, same group [63] compared the performance of this method with that achieved by using asymmetric PCR. The same dynamic range (between 30 and 3000 copies) were obtained with both amplification strategies and similar LODs of 11 and 15 copies (0.4 and 0.5 aM) using PCR and HAD, respectively, were found. The electrochemical genosensor coupled with both amplification approaches demonstrated comparable and successful results in the detection of *M. tuberculosis* in clinical samples (sputum, urine, and pleural fluid samples).

A microfluidic-multiplexed electrochemical platform with integrated sensors composed of CNTs associated with Fc for direct impedimetric detection without any amplification of genomic DNA from *M. tuberculosis* and pathogenic viral DNA from hepatitis C in clinical isolates was developed by Zribi et al. [64]. The monolithic chip integrates three independent fluidic polydimethylsiloxane channels, each containing one electrochemical chamber with three gold electrodes to perform three independent measurements (negative control, DNA detection and DNA mismatch detection).

The resulting fluidic device, due to the flow and geometry, enhanced 50 times the capture rate (from 0.02 to 1 DNA strand per s), six times the electron transfer rate constant (from 1 up to 6 s^−1^) and decreased the LOD of the electrochemical biosensor from picomolar (2 pM) in bulk solution to femtomolar (7 fM). In addition, it offered a large dynamic range (0.1 fM–1 pM) for a synthetic ssDNA from Hepatitis C virus. The miniaturized microfluidic device allowed also direct detection of *M. tuberculosis* (H37Rv) *rpoB* allele in DNA extracted from clinical isolates without PCR amplification.

**Figure 7:**
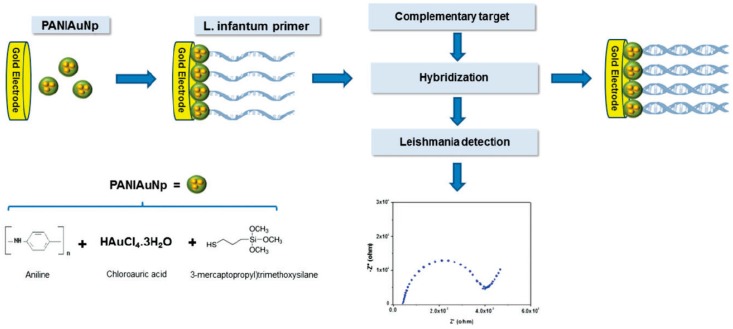
Schematic representation of the impedimetric genosensor developed for *L. infantum* determination. Reprinted from [65] with permission.

García et al. developed an electrochemical DNA sensor for specific detection of *Leishmania infantum (L. infantum)* genome [65]. This biosensor involved the immobilization of a DNA probe onto a gold electrode previously coated with a layer of 3-mercaptopropyltrimethoxysilane on polyaniline matrix containing AuNPs (PANIAuNPs) (Figure 7). By evaluating the primer-genome interaction by EIS using Fe(CN)_6_^3−/4−^, the developed sensor recognized the *L. infantum* genomic DNA at different concentrations (1–4 ng mL^−1^) and demonstrated applicability to the analysis of contaminated canine serum samples.

**Figure 8:**
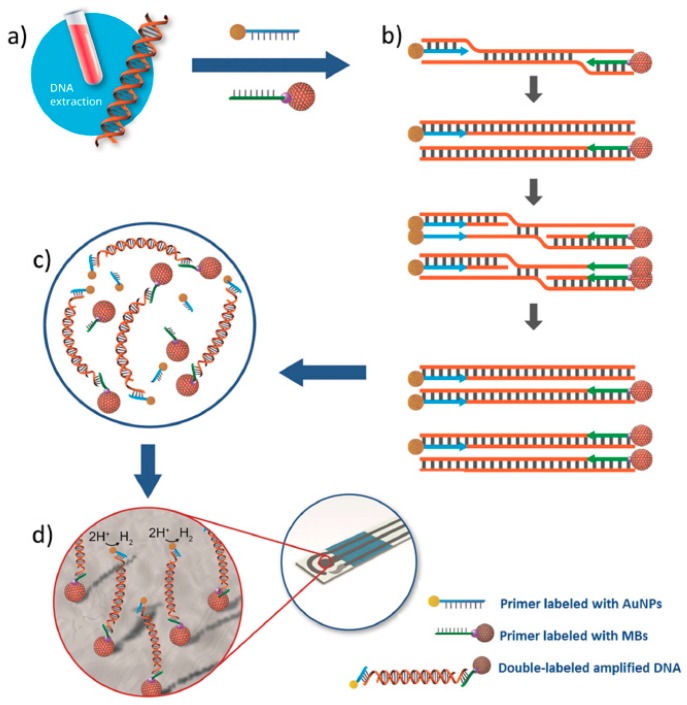
Scheme of the electrochemical method developed to determine *Leishmania* DNA by using RPA and primers labeled with AuNPs and MBs involving the following steps: DNA extracted from dog blood (**a**), isothermal amplification of a specific region by RPA using primers labeled with AuNPs and MBs (**b**), magnetic capture of the MB/amplified DNA/AuNP complexes of the SPCE (**c**) and chronoamperometric detection of the hydrogen evolution reaction (HER) by AuNPs (**d**). Reprinted from [66] with permission.

Other interesting methodology for the determination of *Leishmania* DNA was developed combining the use of isothermal recombinase polymerase amplification (RPA), primers labeled with MBs and AuNPs and electrochemical detection at SPCEs (Figure 8) [66]. After magnetic capture of the double-labeled amplified product (MB/amplified DNA/AuNP complex) onto the SPCE, the chronoamperometric measurement of the electrocatalytic activity of the AuNPs towards the hydrogen evolution reaction allowed the determination of the amplicons. This method exhibited a linear relationship between the measures current and the logarithm of parasite concentration in the range between 0.5 and 500 parasites per mL of blood, a LOD of 0.8 parasites per mL of blood and perfect discrimination between blood samples from healthy and infected dogs.

## 4. General Considerations

This review article sheds useful insights into the latest advances, current trends and prospects in the electrochemical genosensing of circulating biomarkers. The highlighted methods have been developed for the determination of circulating biomarkers associated with cancer (mutations in the *EGFR* and *p53* genes, DNA species related to *ORAOV*1, *BRCA* and *HPV-16* genes, mRNA associated to IL-8 and many cancer related miRNAs), neurodegenerative diseases (PrPC proteins, Alzheimer-related DNA sequences, mutated *apolipoprotein E* gene), bacterial (*E. coli*, *Porphyromonas gingivalis* 16S rRNA, *S. pneumoniae lytA* gene *Mycobacterium* sp. genomic DNA, *M. tuberculosis* repetitive insertion sequence IS6110 and *rpoB* gene, *L. infantum* genomic DNA, *Leishmania* DNA and *S. haematobium* 16S rRNA) and viral infections (DENV-3-specific DNA sequences, Hepatitis C viral DNA, HIV antibodies and human norovirus). In particular, selected contributions describe the development of versatile electrochemical nucleic acid based scaffolds for determining different levels of circulating biomarkers mainly of genetic but also of protein nature. The compiled approaches are based on the use of conventional electrodes (gold, GCE, ITO, PGE), arrays of gold electrodes prepared by photolithography or disposable electrodes (SPCEs, Au-SPEs), different bioreceptors, mainly linear and hairpin DNAs but also RNA probes, aptamers and peptides, amplification strategies (‘‘junction-probe’’ isothermal amplification, nuclease-assisted target recycling, formation of long-range self-assembled DNA nanostructures, RCA, RPA, enzymatic and nanomaterials-based amplification) and a wide variety of electrochemical techniques including (chorono) amperometry, DPV, SWV, EIS, DPV, CV, LSV and ACV. The determinations of the circulating biomarkers with electrochemical genosensors have been performed mainly in serum, saliva, plasma, and urine samples. It is worth mentioning at this point that the choice of an electrochemical technique in the highlighted approaches is closely related with particular variables like the selected electrochemical label and the assay format, as well as, of course, the target analyte and sample matrix. Therefore, although experience in this field could help in some way, the wide variability of available electrochemical techniques makes it difficult to recommend a priori the most appropriate and selection should be made depending on the particular application and only by experimental testing.

By the number of strategies described so far in this field, the determination of circulating biomarkers associated with cancer and bacterial and viral infections have gained special relevance in this field, and the use of sandwich hybridization formats followed by direct approaches both involving DNA linear probes are by far the most common strategies. Other less common but very attractive approaches are based on the use of TRE assays.

In parallel with remarkable progress in nanotechnology and bioconjugation techniques, some of these electrochemical genosensing strategies have been developed onto nanostructured surfaces using graphene, graphene oxide, mesoporous nanomaterials, AuNRs and MWCNTs and/or using bioconjugates of AuNPs with multiple bioreceptors as signaling carriers. Dendrimers such as PAMAN G4 and polymers (PPy, PANI) have been also used also as electrode modifiers to improve the performance of these electrochemical genosensors for circulating biomarkers determination. Moreover, these approaches have been developed both using integrated formats and by coupling conveniently modified MBs with SPEs or magnetic-conventional electrodes.

The developed electrochemical genosensors have demonstrated to detect protein and genetic biomarkers in the 0.5 pM–1 nM and 1 aM–3.09 nM ranges, respectively, depending of each application but all of them have demonstrated to be suitable for practical purposes through the analysis of real samples.

## 5. Challenges to Address and Future Prospects

Nowadays healthcare systems are pursuing the development of cheap, rapid, non-invasive and user-friendly tools able to provide multiple results from a single sample even in remote settings to help in the timely decision-making. Moreover, the use of information and communication technologies in healthcare (e-health) to improve diagnosis, treatment, monitoring and management is also stimulating the research and the development of portable devices for patient-monitoring.

In this sense, electrochemical genosensing has clearly been gaining increasing relevance in the clinical analysis field using non-invasive approaches, such as the determination of circulating biomarkers. The resulting devices can be portable, easy-to-use, with rapid response times, affordable and easily tailored for the needs of low- and middle-income countries.

Although exhaustive literature evaluation shows there is a large number of electrochemical nucleic acid-based biosensors able to comply with the sensitivity and selectivity demanded for practical applications, most of them still remain in the proof of concept or prototype stages of development and only a limited number (those highlighted in this review) have demonstrated applicability in the analysis of real non- or minimally invasive samples (mainly serum, urine and saliva). However, it is worth to mention also that the number of real patient samples used in these methods is never enough to assure a reliable validation of the genosensor and that currently only a very limited number of circulating biomarkers have been clinically validated. The compulsory validation of these electrochemical genosensing devices using large numbers of patient samples must be performed not only in terms of sensitivity, selectivity and accuracy, but also of rapidity, simplicity and cost with respect to other available competitive methodologies.

It is worth to mention in this point that despite the tremendous progress made in the latest years and the very promising capabilities already demonstrated for circulating biomarkers determination, unfortunately the transition of electrochemical genosensors from laboratories to the market is complicated and none has crossed the technological valley of death to successful commercialization. The translation of current research prototypes into commercial products should mainly address some quality assurance challenges. One of the most critical aspects to be solved is the achievement of reproducible quantitative results which depends both of the feasibility of producing identical sensor batches and of the control of environmental variables which can affect significantly their performance. A second aspect to be tackled relates with the difficulty on developing simple-to-use assays for non-laboratory trained individuals, more prone than competent staff in well-run laboratories to making mistakes. 

Moreover, to achieve the commonly accepted usage of electrochemical genosensors for POC testing, additional research efforts are needed toward their full integration in automated and miniaturized systems. Currently, the complicated assembly process of the genosensors and their subsequent scaling up to mass production makes the manufacturing of commercially available devices challenging and expensive. Other challenges crucial for commercialization prospects include ensuring the storage stability of the prepared nucleic acid-based bioplatforms and the bioconjugates of nanomaterials used commonly for amplification purposes and their transportation conditions for appropriate functionality. 

Therefore, further efforts on genosensor stability and validation, together with their continuous miniaturization and automatization, are key steps to ensure their successful use in POC testing for making clinical results available at patient bedside or physician office. One cannot fail to mention that additional challenge in electrochemical genosensing continue being the reliable, simultaneous detection of multiple circulating biomarkers and proper attention to the nonspecific adsorption issues in complex biological samples.

Although the important and complex challenges to be addressed makes it likely that several years will pass before electrochemical genosensing devices will fully replace techniques currently used for circulating biomarker routine determinations, the electrochemical genosensing diagnostic platforms are eliciting considerable excitement as they promise a paradigmatic shift in how disease diagnosis and health monitoring will be conducted in the near future. The development and deployment of these systems could ultimately lead to more rapid clinical decision making and corresponding reductions in patient stress and healthcare costs. Moreover, it is undoubtedly that with constant developments in molecular biology, nanofabrication methods and labeling, nanoinstrumentation, and multiplexing capabilities, rapid, sensitive, selective, and easy-to-use electrochemical genosensors will find an important niche for circulating biomarkers determinations in the clinical field.

## Figures and Tables

**Table 1:** Electrochemical genosensors for determination of cancer-related circulating miRNAs.

Electrode	Approach/Type of Hybridization Assay	Target miRNA/Disease	Electrochemical Technique/Redox Probe	L.R.	LOD	Applicability	Reference
Gold disk electrode	Immobilization of a thiolated DNA probe/Direct competitive and complexation of the biotin-miRNA with Fc-capped AuNPs/streptavidin (Strep) conjugates	miRNA-182	CV/Fc	10 fM–2.0 pM	10 fM	Sera of glioma patients	[31]
Magnetic-controllable gold electrode	Sandwich hybridization, ‘‘junction-probe’’ isothermal amplification strategy and MBs-based enzymatic amplification	hsa-miR-200a	Chronoamperometry/TMB + H_2_O_2_	1 aM–10 fM	0.22 aM	Spiked saliva samples	[39]
AuNPs-modified SPCE	Immobilization of a thiolated RNA probe/Direct hybridization (1), p. 19 binding onto the RNA-RNA duplex formed on the electrode surface (2) and displacement of the p19 attached to the electrode by incubation in a mixture of a target miRNA and a nonthiolated RNA probe at high concentration	miRNA-21, miRNA-32, and miRNA-122	SWV/K_3_[Fe(CN)_6_] and [Ru(NH_3_)_6_]Cl_3_	10 aM–1 μM	5 aM	Human serum samples	[33]
Gold disk electrode	Immobilization of a thiolated DNA probe together with thioglycolic acid, direct hybridization and isothermal amplification by a DSN	miRNA let-7b	EIS/[Fe(CN)_6_]^4−/3−^	2.0 fM–2.0 pM	1.0 fM	Human serum samples	[40]
Three-electrode biosensor fabricated on a polystyrene substrate	Immobilization of a thiolated DNA probe (probe 1), MCH and BSA/Direct hybridization and RCA amplification using a mixture of the target miRNA, probe 2 (DNA added for initiation of RCA amplification), a cyclized padlock probe and phi29 DNA polymerase	miRNA-143/Cancer	Chronocoulometry/Ruhex	100 fM–1 nM	100 fM	Spiked human blood samples	[41]
Gold disk electrode	Immobilization of a molecular beacon, sandwich hybridization and mediated SDA (using Klenow fragment (3′–5′exo) and Nb.BbvCI nicking exonuclease) and enzymatic amplifications (Strep-AP)	miRNA-222	DPV/α-NP	50 pM–10 nM	40 pM	Spiked human serum samples	[42]
Au-SPEs	Immobilization of a thiolated RNA probe/Direct	miRNA-155/	SWV/[Fe(CN)_6_]^3−/4−^	10 aM–1.0 nM	5.7 aM	Human serum samples	[29]
GCE functionalized with AuNRs decorated on GO sheets	Immobilization of a thiolated RNA probe/Direct	miRNA-155	DPV/Oracet Blue (OB)	2.0 fM–8.0 pM	0.6 fM	Spiked human plasma samples	[30]
Magnetic-GCE	DSNATR and biotinylated capture probes enriched from the solution to the electrode surface using MBs	miRNA-21	EIS/[Fe(CN)_6_]^4−/3−^	—	60 aM	Human serum samples	[43]
GCE	target recycling, nicking-replication reaction and DNAzyme catalysis coupling	miRNA-21	Amperometry/TMB + H_2_O_2_	1 aM–100 pM	0.5 aM	Spiked human serum samples	[44]

Abbreviations: AuNPs: gold nanoparticles; AuNRs: gold nanorods; CV: cyclic voltammetry; DNS: duplex specific nuclease; DPV: differential pulse voltammetry; DSNATR: duplex-specific nuclease assisted target recycling; EIS: electrochemical impedance spectroscopy; Fc: ferrocene; GO: graphene oxide; SDA: strand displacement amplification; SWV: square wave voltammetry; TMB: 3,3′,5,5′ tetramethylbenzidine.

## Abbreviations

ACV	alternating current voltammetry
AP	alkaline phosphatase
AP1, AP2	auxiliary probes
aPCR	asymmetric PCR
APTMS	(3-aminopropyl)trimethoxysilane
AuNPs	gold nanoparticles
AuNRs	gold nanorods
CFU	colony forming unit
CPEG	*O*-(3-carboxypropyl)-*O*′-[2-(3-mercaptopropionyl-amino)ethyl]-polyethylene glycol
CPs	conducting polymers
CV	cyclic voltammetry
daPCR	direct asymmetric PCR
DENV-3	dengue virus serotype 3
DNS	duplex specific nuclease
DPV	differential pulse voltammetry
ds	double-stranded
DSNATR	duplex-specific nuclease assisted target recycling
EDC	1-ethyl-3-(3-dimethylaminopropyl)carbodiimide
EGFR	epidermal growth factor receptor
EIS	electrochemical impedance spectroscopy
ELISAs	enzyme linked immunosorbent assays
Fc	ferrocene
FITC	fluorescein isothiocyanate
GCE	glassy carbon electrode
GO	graphene oxide
GSHs	graphene and mesoporous hybrid nanomaterials
HDA	helicase-dependent DNA amplification
HER	hydrogen evolution reaction
HIV	human immunodeficiency virus
HPV	human papillomavirus
HQ	hydroquinone
ITO	indium-tin oxide
LODs	detection limits
LSV	linear sweep voltammetry
MB	methylene blue
MBs	magnetic beads
MCH	6-mercaptohexanol
MSGNs	graphene and mesoporous multifunctional hybrids materials
MWCNTs	multi-walled carbon nanotubes
NHS	N-hydroxysuccinimide
NSCLC	non-small cell lung carcinoma
PAMAM	polyamidoamine
PANI	polyaniline
PCR	polymerase chain reaction
Pg	*Porphyromonas gingivalis*
PGE	pencil graphite electrode
POC	point-of-care
POCT	POC tests
PrPC	human cellular prions
Py	pyrrole
RPA	isothermal recombinase polymerase amplification
SAM	self-assembled monolayer
SDA	strand displacement amplification
Sp	signaling probe
SPEs	screen-printed electrodes
SPCEs	screen-printed carbon electrodes
ss	single-stranded
Strep-HRP	streptavidin-peroxidase
SWV	square wave voltammetry
TMB	3,3′,5,5′ tetramethylbenzidine
TSEs	transmissible spongiform encephalopathies
TTF	tetrathiafulvalene
